# Longitudinal impact of Intersocietal Accreditation Commission vein treatment center accreditation on practice patterns, safety metrics, and patient outcomes

**DOI:** 10.1016/j.jvsv.2025.102315

**Published:** 2025-09-15

**Authors:** Fedor Lurie, Mohamed Osman, Marlin Schul, Jeffery P. Schoonover, Kelly Hallett, Mary Beth Farrell, Mark H. Meissner

**Affiliations:** a Jobst Vascular Institute, Toledo, OH; b Division of Vascular Surgery, University of Michigan, Ann Arbor, MI; c Indiana Vascular Associates, Lafayette, IN; d Indiana Vein & Lymphatic, Carmel and Fishers, IN; e Molecular Imaging Services, Pheonix, AZ; f Department of Surgery, University of Washington School of Medicine, Seattle, WA

**Keywords:** Accreditation, Venous disease, Quality improvement, Vein centers, Patient outcomes, Practice standards

## Abstract

**Objective:**

With health care rapidly expanding and patient accessibility needs increasing, there has been an influx of providers often lacking formal training in venous disease management. The aim of this study was to determine if centers that participate in an accreditation program exhibit increased quality, safety outcomes, and overall practice standards.

**Methods:**

Of 325 accredited vein centers, 287 underwent reaccreditation within 3 years. Fifty-nine of them were compliant with Intersocietal Accreditation Commission (IAC) standards at the time of initial accreditation. Fifty-nine IAC-accredited centers participated in the American Vein and Lymphatic Society Pro Vein registry and had patient-level data. Sixteen were initially compliant with IAC standards (group 1; 4977 patients) and 43 had deficiencies (group 2; 11,179 patients). A stratified before-and-after design was used to analyze center-level and patient-level data (demographics, body mass index, and disease severity scores [Clinical-Etiological-Anatomical-Pathophysiological and revised Venous Clinical Severity Score (VCSS)]. Primary outcomes included compliance with IAC standards, treatment results (eg, VCSS changes, complications, endothermal heat-induced thrombosis >2), and interventional practice patterns, such as intervention rate and Utilization Index.

**Results:**

Of the 287 IAC-accredited vein centers who pursued reaccreditation, 59 were compliant initially and at reaccreditation. The remaining centers (n = 229) had multiple deficiencies, with safety issues persisting in some centers at reaccreditation. Before accreditation, group 2 centers treated younger, lower body mass index patients with less severe disease, and group 1 centers saw more advanced cases. Over time, group 2 centers began treating more severe cases. Group 1 had higher intervention rates and lower use indices before accreditation. Post-treatment complication and endothermal heat-induced thrombosis rates were low and similar across both groups. Group 1 showed a greater VCSS score change after treatment, partly owing to higher baseline scores. Over time, group 2 showed a decrease in Utilization Index, without a post-treatment decrease in the revised VCSS change aligning with group 1, indicating improved practice patterns after accreditation.

**Conclusions:**

IAC accreditation plays a meaningful role in standardizing and improving the quality of outpatient venous care. It promotes safer procedural environments, encourages more selective use of interventions, and is associated with improved clinical outcomes—particularly among initially noncompliant centers. These findings support the expansion of accreditation programs and underscore their importance in maintaining high standards of care in an increasingly heterogeneous field.

**Article Highlights**

• **Type of Research:** Before-after parallel group retrospective review of prospectively collected administrative and registry data
• **Key Findings:** Accreditation of vein centers differentially changes practice pattern and treatment outcomes.
• **Take Home Message:** Outpatient vein practices initially not compliant with Intersocietal Accreditation Commission standards improve practice patterns, safety metrics, and treatment outcomes after completing accreditation.

Chronic venous disease is highly prevalent in Western populations, affecting more than 25 million adults in the United States, with 6 million having advanced disease significantly diminishing patient functional quality of life.^1^^,^^2^ The majority of chronic venous disease patients require superficial veins treatment that sufficiently improve their quality of life.^3^ This treatment option has undergone a profound transformation over the past two decades, shifting from hospital-based surgical procedures to outpatient office-based interventions. Although this transition has increased accessibility and convenience for patients, it has also led to decreased oversight and minimal standardization across practices. Compounding the issue, the field has seen an influx of providers from diverse medical backgrounds, many of whom lack formal training in venous disease management. In some cases, training is limited to brief industry-sponsored workshops, raising concerns about the quality and safety of care provided in this rapidly expanding space.

In response to these concerns, professional societies have adopted various strategies aimed at improving the quality of venous care. These strategies fall into three broad categories: educational, descriptive, and prescriptive approaches. Although there are an estimated 10,000 providers in the United States treating superficial veins,^4^^,^^5^ only a small fraction of them attends venous meetings or pursues certification in venous disease. Clinical practice guidelines provide suggestions based on evidence. Despite their great educational value, they are not actionable, and compliance with their recommendations is variable, and generally low.^6^

Descriptive efforts, such as the Vascular Quality Initiative of the Society for Vascular Surgery Patient Safety Organization and other venous registries, have contributed valuable data on clinical outcomes and quality indicators in venous care.^7^ However, these registries often lack critical information on structural and technical safety aspects—such as sterilization practices, facility standards, and provider qualifications—that are necessary for a comprehensive understanding of quality metrics and patient outcomes.

Prescriptive strategies, such as accreditation, aim to address these gaps. Accreditation is defined as “public recognition by a national health care accreditation body of the achievement of accreditation standards by a healthcare organization, demonstrated through an independent external peer assessment of that organization's level of performance in relation to the standards.”^8^ Accreditation programs, such as those offered by the Intersocietal Accreditation Commission (IAC), establish actionable, enforceable standards for practice. Compliance is monitored through structured application processes and site visits, ensuring that accredited centers meet defined safety and operational benchmarks. However, these standards currently do not include requirements for clinical outcomes of interventions.

Both descriptive and prescriptive initiatives aim to enhance quality, though neither explicitly defines what quality entails. The Institute of Medicine defines health care quality as “the degree to which health services for individuals and populations increase the likelihood of desired health outcomes and are consistent with current professional knowledge,”^9^ emphasizing both safety and effectiveness as central dimensions of quality. Whereas previous studies have explored the relationship between accreditation and safety outcomes,^10^ there remains a paucity of evidence regarding its effect on the clinical effectiveness of venous interventions. Furthermore, direct comparisons between accredited and nonaccredited centers are confounded by selection bias, because accreditation remains a voluntary process, often pursued by more motivated or better-resourced practices.^11^

This study aims to fill this knowledge gap by evaluating the longitudinal impact of IAC Vein Center Accreditation on outpatient venous practices. Specifically, it investigates changes in practice patterns, safety metrics, and patient outcomes, comparing centers that were compliant with IAC standards at the time of application with those that were not but became compliant during the accreditation process.

## Methods

This study was designed as a stratified before-after analysis of prospectively systematically collected practice-level and patient-level data. Included outpatient vein centers were stratified into two groups: centers that were compliant with the IAC standards at the time of application for accreditation (compliant group) and centers that were not compliant with the standards and were brought to compliance during the accreditation process. The accreditation process included review of the application and supporting documentation, followed by a site visit with verification of compliance with IAC standards. Compliance is determined by a panel of representatives of professional societies including the American Venous Forum, American Vein and Lymphatic Society (AVLS), American College of Surgeons, Society for Clinical Vascular Surgery, Society of Interventional Radiology, Society for Vascular Medicine, and Society for Vascular Surgery. Administrative data from both phases of the accreditation process were used for analysis.

Patient-level data were obtained from the AVLS PRO Vein registry as described in a previous publication.^7^ Registry data did not contain patient identifiers and therefore was approved as exempt by the institutional review board. Data extracted from the registry were indexed by provider and combined into a single center for centers with multiple providers. Extraction was performed in three time intervals of reference: 1 to 3 months before IAC application, 6 to 12 months after accreditation, and 20 to 36 months after accreditation (corresponding with the time of applying for reaccreditation). Patient data included demographics, body mass index, clinical class of the Clinical-Etiological-Anatomical-Pathophysiological classification,^12^ and revised Venous Clinical Severity Score (rVCSS).^13^ An initial attempt to extract or calculate the VEINES/Sym score^14^ was unsuccessful owing to the lack of pertinent data in the registry. The outcomes of interest included metrics related to compliance with IAC standards (provider and staff qualifications, noninvasive vascular testing, documentation, and safety), treatment outcomes available for extraction from the registry (change in VCSS score after treatment, incidence of complications, and incidence of endothermal heat-induced thrombosis [EHIT] >2), and practice interventional patterns (intervention rate and Utilization Index). The intervention rate was calculated as the percentage of patients entering the practice who receive a surgical intervention during each time interval. The Utilization Index was calculated as the number of procedure-based visits per patient per year. The rate of complications was defined as an incidence of any complication during 3 months from an intervention.

### Statistical analyses

A descriptive analysis was performed to compare the baseline characteristics of patients treated in both groups of centers. All continuous variables were tested for normality of distribution before selection of the statistics to be used. The results are reported as mean ± standard deviation and as percentage for frequencies. Comparisons between groups were done by using one-way analysis of variance, *t* test for continuous variables, χ^2^ test, or Fisher's exact test as appropriate. The change over time for each group was analyzed by using the general linear model for repeated measures in SPSS (ie, repeated measures analysis of variance). Data was analyzed using SPSS Statistics, version 27.0 (IBM Corp). Sample size calculations were not possible owing to a lack of information on the variability of outcomes in the sample.

## Results

The results of application review and site visits of 325 accredited vein centers were available for analysis. Among them, 287 underwent reaccreditation within 3 years from initial accreditation. Fifty-nine centers were compliant with IAC standards at the time of initial accreditation, and all remained as such at the time of reaccreditation. The remaining 229 centers had multiple issues at the time of initial application (Table I), with all the main issues corrected during the accreditation process. At the time of application for reaccreditation 3 years later, a significant proportion of centers remained compliant with IAC standards. However, some issues, especially safety, remained prevalent (Table I).

**Table I:** Issues identified at initial accreditation and reaccreditation percentages of centers identified with issues at initial accreditation and reaccreditation

Issues	Centers with deficiencies		*P* value (Fisher's exact test)
	Initial accreditation	Reaccreditation	
Staff related issues	51	26	<.01
Insufficient Staff qualifications	7	6	.3
Insufficient volume per provider	20	0.4	<.01
Duplex-related issues	43	7	<.01
No accredited RVTs	25	6	.01
Missing duplex images	34	0	<.01
Protocol issues	15	13	.6
Patient consent issues	42	24	.01
H&P issues	11	8	.5
Not documented CEAP and VCSS	27	16	.01
Patient instructions issues	15	12	.4
Safety issues	52	36	.01
Emergency cart issues	20	18	.6
Infection control	17	16	.5
Autoclave-related	5	5	1
Sterilization issues	9	8	.9

*CEAP,* Clinical-Etiological-Anatomical-Pathophysiological; *H&P,* History and physical examination; *RVT,* Registered Vascular Technologist; *VCSS,* Venous Clinical Severity Score.

A total of 59 IAC-accredited centers that participated in the AVLS Pro Vein registry and had patient data at the time before their accreditation and at least at one of the two follow-up periods were included in this study. Of the 59 practices, 16 were initially compliant with IAC standards (group 1) and 43 had deficiencies (group2).

Patient-level data were available for 4977 patients in group 1, and 11,179 patients in group 2, including preaccreditation time (1112 patients in group 1 and 3421 in group 2), 6 to 12 months after accreditation (2018 in group 1 and 5146 in group 2), and 20 to 36 months after accreditation (1847 in group 1 and 2612 in group 2)

Before accreditation, patient populations treated in IAC-compliant and IAC-noncompliant groups were substantially different. Patients treated in the group 2 centers were younger with a lower body mass index and with less severe disease (Table II). Group 2 centers treated a greater proportion of C2 patients (*P* < .001) and a smaller proportion of C6 patients (*P* < .001) compared with group 1 centers (Fig). The patient population treated in group 1 centers did not change significantly over time. In contrast, patients treated in the group 2 centers had more severe disease at the time after initial accreditation and 20 to 36 months later (Table II). However, the VCSSs in the group 2 remain significantly different from group 1 in all time periods (Table II).

**Table II:** Patients of Intersocietal Accreditation Commission (IAC)-compliant (group 1) and IAC-noncompliant (group 2) venous centers

	Before accreditation			6-12 Months after accreditation			20-36 Months after accreditation			Change over time (*P* value)	
	Group 1	Group 2	*P* value	Group 1	Group 2	*P* value	Group 1	Group 2	*P* value	Group 1	Group 2
Age	55.88 ± 8.49	49.60 ± 8.17	.012	55.88 ± 8.49	52.09 ± 8.61	.511	66.12 ± 5.50	55.88 ± 8.49	.123	1.000	.055
Female sex	65.9 (5.63)	69.12 (10.69)	.255	64.13 (5.06)	65.21 (5.78)	.138	63.38 (3.63)	67.14 (5.76)	.018	.267	<.001
BMI	31.29 ± 3.29	28.45 ± 2.95	.002	30.94± 2.73	28.96 ± 3.02	.026	31.84 ± 3.30	29.84 ± 2.87	.026	.248	.140
CVI (%)	47.56 (7.61)	36.58 (7.84)	<.001	49.31 (6.88)	41.37 (10.49)	.007	50.13 (6.96)	42.63 (10.48)	.011	.278	<.001
rVCSS	9.3 ± 5.9	4.0 ± 3.4	<.001	9.2 ± 6.1	6.8 ± 2.7	.04	10.3 ± 5.2	6.4 ± 2.0	<.001	.796	<.001

*BMI,* Body mass index; *CVI,* chronic venous insufficiency; *rVCSS,* revised Venous Clinical Severity Score.

Values are mean ± standard deviation or number (%).

**Fig:**
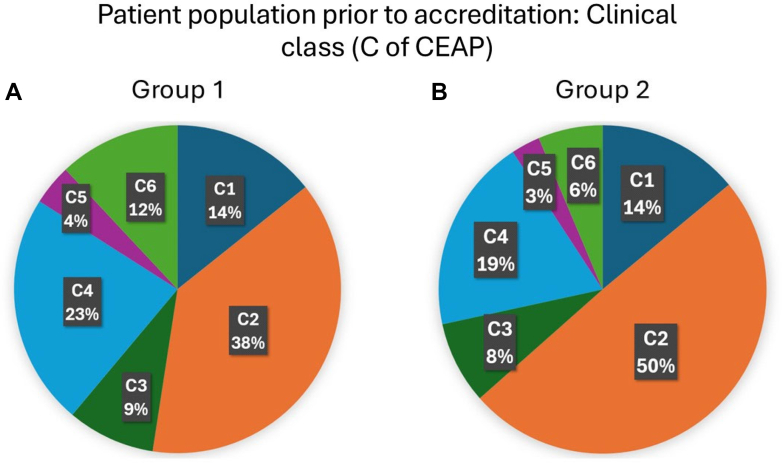
Distribution of the clinical class (C of the Clinical-Etiological-Anatomical-Pathophysiological classification) among patients in Intersocietal Accreditation Commission (IAC)-compliant and IAC-noncompliant centers before accreditation. Pie chart representations of **(A)** group 1 and **(B)** group 2 patient populations before accreditation by clinical classification.

Before accreditation, group 1 centers had a higher intervention rate and lower Utilization Index compared with group 2 centers (Table III). The incidence of post-treatment complications and EHIT was not different between the two groups. Patients in group 1 had a large change in VCSS score after treatment, reflecting at least in part a higher baseline VCSS value. There were no significant changes in the treatment outcomes or interventional patterns over time in the group 1 (Table III). Among the group 2 centers, the incidences of complications and EHIT also did not change, remaining at very low levels. There was a small but statistically significant increase in VCSS score change after treatment in the group 2 centers. The most substantial postaccreditation change was observed in the interventional pattern in group 2 centers, with significant decrease in the Utilization Index, which became not different from group 1 (Table III).

**Table III:** Treatment outcomes and interventional patterns of Intersocietal Accreditation Commission (IAC)-compliant (group 1) and IAC-noncompliant (group 2) venous centers

	Before accreditation			6-12 Months after accreditation			20-36 Months after accreditation			Change over time (*P* value)	
	Group 1	Group 2	*P* value	Group 1	Group 2	*P* value	Group 1	Group 2	*P* value	Group 1	Group 2
rVCSS change	4.6 ± 1.6	2.8 ± 1.2	<.001	6.1 ± 5.3	3.6 ± 1.5	.006	6.6 ± 5.3	3.2 ± 2.2516	.001	.418	<.001
Complications (%)	0.53 (0.37)	0.47 (0.29)	.519	0.44 (0.31)	0.42 (0.23)	.784	0.54 (0.32)	0.43 (0.24)	.184	.692	.7
EHIT >2	0.15 (0.26)	0.18 (0.26)	.656	0.23 (0.23)	0.18 (0.26)	.575	0.31 (0.29)	0.20 (0.27)	.21	.248	.9
Intervention rate	0.52 (0.17)	0.46 (0.16)	.016	0.51 (0.13)	0.49 (0.13)	.211	0.51 (0.18)	0.49 (0.15)	.4	.9	.05
Utilization Index	1.51 (0.18)	1.79 (0.29)	.001	1.51 (0.18)	1.50 (0.19)	.6	1.50 (0.13)	1.51 (0.21)	.76	.97	.002

*EHIT,* Endothermal heat-induced thrombosis; *CVI,* chronic venous insufficiency; *rVCSS,* revised Venous Clinical Severity Score.

Values are mean ± standard deviation or number (%).

## Discussion

The primary finding of this study is the differential impact of IAC accreditation on outpatient vein centers, depending on their initial compliance with IAC standards. Centers that were already compliant at the time of application (group 1) maintained consistently high standards over time, with stable patient demographics, consistent interventional patterns, and unchanged treatment outcomes after accreditation. In contrast, centers that were not compliant at baseline (group 2) demonstrated significant improvements in their adherence to standards, interventional behaviors, and clinical outcomes after accreditation. Although these standards do not directly mandate clinical outcomes, this study suggests that their implementation is associated with improved resource use and better patient outcomes, as reflected in greater rVCSS improvement and decreased procedure frequency per patient.

The IAC standards, created by a multisocietal team of experts, reflect minimal requirements for provider and staff qualifications, patient documentation, and practice settings related to patient safety, infection control, and standard operational procedures. At the time of the study, only 325 vein centers were accredited by the IAC, likely representing a small proportion of venous practices in the United States. The finding that, even among vein centers that apply for accreditation, noncompliance with these minimal requirements is exceptionally high is alarming. More than one-half of these centers had unsatisfactory staff qualifications, 52.2% had significant patient safety issues, 41.9% had deficiencies in consenting patients, and every fourth center had an ultrasound examination performed by an unqualified person.

These findings are particularly striking in group 2 centers, which initially treated a younger, less severely affected patient population but performed more procedures per patient. Despite lower baseline disease severity, these centers had a higher Utilization Index compared with group 1 centers. This discrepancy raises concerns about overuse and suboptimal patient selection in settings lacking external oversight. After accreditation, group 2 centers demonstrated a marked decrease in their Utilization Index, aligning with group 1, while simultaneously showing a greater improvement in post-treatment rVCSS scores. This finding suggests that accreditation fosters more appropriate use of interventions and higher-quality care delivery.

Moreover, accreditation appears to influence not just structural and procedural metrics, but also clinical decision-making. Over time, group 2 centers showed a shift in their patient population toward more advanced clinical presentations (chronic venous insufficiency) and higher baseline severity scores. These changes, coupled with improved outcomes and reduced intervention frequency, suggest that accreditation may contribute to more effective triaging and treatment strategies, even in initially underperforming practices. Another important insight is the sustainability of compliance. Although most group 2 centers retained their accredited status at reaccreditation, some safety-related lapses reemerged. This finding highlights the need for ongoing monitoring and possibly interim evaluations between accreditation cycles to ensure continued adherence to safety and quality benchmarks.

Despite its strengths, this study has limitations. It is inherently biased toward centers that opted into the accreditation process and participated in the AVLS PRO Vein Registry, which may not be representative of the broader landscape of vein treatment practices. Nonaccredited centers may differ in terms of provider background, case mix, or procedural volume. However, it is unlikely that vein practices that have not pursued accreditation are significantly different from group 2. A previous study showed that the differences between IAC accredited, and IAC nonaccredited centers are similar to those between groups 1 and 2 found in this study.^11^ For example, patients treated at nonaccredited centers were significantly less likely to present with chronic venous insufficiency, most commonly presented with varicosities, and had lower VCSSs. The use of endovenous ablation was significantly higher in nonaccredited centers (2.03 vs 1.51; *P* < .001). Additionally, as an observational study, causality cannot be established definitively, and unmeasured confounding variables may have contributed to observed improvements. However, the magnitude and consistency of changes across multiple domains and time intervals provide compelling support for the accreditation's positive impact.

## Conclusions

IAC accreditation plays a meaningful role in standardizing and improving the quality of outpatient venous care. It promotes safer procedural environments, encourages more selective use of interventions, and is associated with improved clinical outcomes—particularly among initially noncompliant centers. These findings support the expansion of accreditation programs and underscore their importance in maintaining high standards of care in an increasingly heterogeneous field.

## Author Contributions

Conception and design: FL, JS, MM

Analysis and interpretation: FL, MO, MF, MM

Data collection: FL, MS, KH, MF

Writing the article: FL, MO, JS, KH, MM

Critical revision of the article: FL, MO, MS, JS, KH, MF, MM

Final approval of the article: FL, MO, MS, JS, KH, MF, MM

Statistical analysis: MF, FL

Obtained funding: FL

Overall responsibility: FL

## Funding

Grant funding was provided by the Intersocietal Accreditation Commission (IAC).

## Disclosures

None.
